# Amylin inhibits gastric cancer progression by targeting CCN1 and affecting the PI3K/AKT signalling pathway

**DOI:** 10.1080/07853890.2025.2480754

**Published:** 2025-03-31

**Authors:** Li Liu, Wenxuan Liu, Wenhong Deng

**Affiliations:** Department of General Surgery, Renmin Hospital of Wuhan University, Wuhan, Hubei Province, China

**Keywords:** Amylin, gastric cancer, CCN1, transcriptome sequencing, EMT

## Abstract

**Background: **After glucagon and insulin, amylin is the third most significant and active islet hormone. Amylin and insulin act synergistically to suppress postprandial blood glucose levels. Research has shown that insulin plays a vital role in the growth and progression of cancer; however, the relationship between amylin and gastric cancer (GC) is unclear.

**Methods:**

This study used a combination of *in vitro* and *in vivo* experiments to investigate the role of amylin in the progression of GC. The expression of amylin in GC and its clinical correlation were evaluated using 38 pairs of GC and healthy human clinical samples. *In vitro* studies, human GC cell lines were treated with amylin to evaluate the effects of amylin on the proliferation, apoptosis and migration of GC cells. In *in vivo* studies, xenograft mouse models were established by subcutaneous injection of GC cells into nude mice, followed by treatment with amylin to assess tumor growth. Finally, Next-Generation Sequencing Technology (RNA-seq) was used to explore the potential mechanism of amylin on GC.

**Results:**

We found that amylin expression was reduced in GC compared to adjacent normal gastric tissues and that elevated amylin expression was negatively correlated with adverse pathological factors (*p* < 0.05). Additionally, we demonstrated that amylin impeded the growth, invasion, migration, and colony formation of GC cells and suppressed the epithelial-to-mesenchymal transformation of these cells (*p* < 0.05). Tumour xenograft model experiments confirmed the tumour-suppressive effect of amylin in subcutaneous tumours in nude mice (*p* < 0.05). Transcriptome sequencing (RNA-seq) revealed that amylin significantly down-regulated CCN1 gene expression in GC cells (*p* < 0.001). Further intervention targeting CCN1 verified its significance as a target of amylin’s anti-carcinogenic function in GC. Additionally, Kyoto Encyclopedia of Genes and Genomes (KEGG) pathway enrichment analysis revealed that amylin exerted its oncogenic effects by inhibiting the PI3K/Akt signalling pathway (*p* < 0.05).

**Conclusions:**

Our findings demonstrate that amylin plays a crucial role in suppressing gastric cancer progression by targeting CCN1 and inhibiting the PI3K/Akt signalling pathway. These results suggest that amylin could serve as a potential therapeutic agent for GC treatment.

## 1. Introduction

Gastric cancer (GC) is one of the most prevalent malignant tumours worldwide. The annual incidence is projected to exceed 10,000 new cases, making it the fifth most common malignant tumour diagnosed globally. Due to its high mortality rate and late-stage detection, gastric cancer is the third leading cause of cancer-related deaths [1]. Several etiologic factors contribute to gastric cancer, including dietary factors such as high salt intake, smoking, and *Helicobacter pylori* (*H. pylori*) infection [2]. A profound deficiency in understanding the molecular pathways involved in the genesis and progression of gastric cancer has resulted in a lack of effective late-stage treatment options. Radical surgery remains the only treatment for localized disease, but the majority of patients are diagnosed at an advanced stage [3]. Median overall survival for advanced gastric cancer is less than one year, even with multiagent chemotherapy [4]. Therefore, identifying novel pharmacological targets and gaining a deeper understanding of the mechanisms driving GC progression are imperative.

Gastric cancer cells exhibit increased glucose intake and distinct glucose metabolism compared to normal epithelial cells. Enhanced aerobic glycolysis (the Warburg effect) in gastric cancer supports cell growth [5]. Amylin is a crucial glucose-regulating hormone [6]. Amylin, the third most significant islet hormone after glucagon and insulin, is co-secreted with insulin to lower blood glucose levels postprandially [7]. Pancreatic islet β-cells primarily synthesize Amylin, but it is also expressed in the neurological system, lung tissue, and gastrointestinal tract [8]. Discovered in 1986, Amylin has been extensively studied, leading to the development of the diabetic medication pramlintide, an Amylin analogue used to treat both type 1 and type 2 diabetes mellitus [9,10]. In the field of oncology, previous studies have found that soluble factors in pancreatic cancer cells selectively stimulate the secretion of Amylin from pancreatic islet cells and that elevated Amylin concentrations are an early feature of pancreatic cancer, so characterization and measurement of pancreatic cancer-derived Amylin may help in the early detection of this disease [11,12]^.^ While Amylin is essential for cancer development, the relationship between Amylin and GC remains unclear. Research on Amylin provides new insights into the genesis and progression of gastric cancer, offering a novel approach to identifying biomarkers and potential therapeutic targets for the disease.

Cellular communication network factor 1 (CCN1), formerly known as CYR61, is a secreted stromal cell protein dynamically expressed in various contexts [13]. CCN1 mediates numerous complex processes during embryonic development and in pathological situations, including fibroblast, endothelial, and mesenchymal cell chemotaxis, growth factor-induced DNA synthesis, cell survival, and angiogenesis [14]. In cancer cells, CCN1 not only induces potent angiogenesis but also enhances cell survival, invasion, and metastasis when overexpressed [15]. Recent studies suggest that CCN1 promotes the growth and infiltration of gastric cancer cells and accelerates the malignancy of gastric cancer [16,17]. One well-researched intracellular signalling pathway is the PI3K/AKT pathway, which is a critical regulator of cell survival, proliferation, and metabolism, and its dysregulation is frequently observed in GC. Activation of this pathway promotes tumour growth, invasion, and resistance to therapy, making it a key target for GC treatment. PI3K/AKT signalling interacts with multiple downstream effectors, including CCN1, Recent research indicates that CCN1 stimulates the expression of PI3K/AKT in various cancer cell types, including glioma, breast, gastric, and renal cancer cells [18–20].

In this study, we hypothesized that Amylin’s effects on the PI3K-Akt signalling pathway, which it targets *via* the CCN1 gene, might inhibit the progression of gastric cancer cells. We examined the expression levels of Amylin in gastric cancer patients and its impact on the invasion, migration, proliferation, and apoptosis of gastric cancer cells to validate this hypothesis. Additionally, we explored the downstream signalling mechanisms of Amylin using Next-Generation Sequencing Technology (RNA-seq).

## 2. Materials and methods

### 2.1. Clinical samples

The study population included 38 gastric cancer (GC) patients who underwent surgical resection at Renmin Hospital of Wuhan University between 2021 and 2022. Serum samples and paired tumour and adjacent normal tissues were collected from each patient. The inclusion criteria were as follows: (1) histologically confirmed gastric adenocarcinoma, and (2) no prior history of chemotherapy or radiotherapy. The exclusion criteria included: (1) the presence of other malignancies, and (2) incomplete clinical data. Stages of gastric cancer were determined based on the TNM classification criteria of the eighth edition of the AJCC (American Joint Committee on Cancer). All patients gave their written, informed permission prior to surgery for data analysis. This study was conducted in accordance with the Declaration of Helsinki and International Conference on Harmonization Guidelines for Good Clinical Practice. This study was approved by the Ethics Institutional Review Board of Renmin Hospital of Wuhan University (WDRY2022-K258).

### 2.2. Cell culture and reagents

The human gastric cancer cell lines HGC-27, AGS, MKN-45, BGC-823, and the gastric normal epithelial cell line GES-1 were obtained from the Chinese Culture Collection Center of Typical Cultures (CCTCC, Wuhan, China). Cells were cultured in RPMI 1640 medium (catalogue #SH30809.01, Hyclone, Logan, TX, USA) at 37 °C in a humidified atmosphere containing 5% CO2. The medium was supplemented with 10% foetal bovine serum (catalogue #10437-028, Gibco, NY, USA) and 1% penicillin/streptomycin (catalogue #SV30010, Hyclone). For functional assays, cells were treated with recombinant human amylin or vehicle control (PBS). Amylin (catalogue #HY-P1059, PHOENIX BIOTECH, USA) was purchased for use in experiments. The primary antibodies used in this investigation were: Amylin (catalogue #PA5- 84142, Invitrogen), CALCR (catalogue #ab313335, Abcam), CCN1 (catalogue #ab228592, Abcam), GAPDH (catalogue #ab9485, Abcam), PI3K (catalogue #4249S, Cell Signaling Technology), P-PI3K (catalogue #4228S, Cell Signaling Technology), AKT (catalogue #9272S, Cell Signaling Technology), P-AKT (catalogue #4060S, Cell Signaling Technology), mTOR (catalogue #2972S, Cell Signaling Technology), P-mTOR (catalogue #5536S, Cell Signaling Technology). For the protein markers involved in cell adhesion and signalling pathways, the following antibodies were used:E-cadherin (catalogue #14472S, Cell Signaling Technology), N-cadherin (catalogue #13116S, Cell Signaling Technology), Vimentin (catalogue #5741S, Cell Signaling Technology), Snail-2 (catalogue #3879S, Cell Signaling Technology),β-catenin (catalogue #9582S, Cell Signaling Technology).

### 2.3. Cell transfection

The pCMV6 plasmids used for transfection contained the human open reading frames CCN1 (NM_001554). Additionally, the plasmids also carried pRS containing short hairpin RNA (shRNA) targeting CCN1. As controls, either pCMV6 without an insert or pRS with negative control shRNA were utilized (Origene, Rockville, MD, USA). Following the manufacturer’s instructions, every cell transfection was carried out in Lipofectamine-2000 (Invitrogen, Carlsbad, CA, USA). Neomycin was used for pCMV6 and puromycin for pRS in the selection of stable transfectants (Invitrogen). The cells were examined using a fluorescence microscope, and the transfection efficacy of CCN1 in GC cells was assessed using quantitative real-time PCR.

### 2.4. Fluorescence quantitative real-time PCR

The RNA extraction from cells or tissues was performed using Trizol reagent. The RNA was converted into complementary DNA (cDNA) using a reverse transcription-polymerase chain reaction (RT-PCR) kit manufactured by Thermo Fisher Scientific. SYBR Green PCR Reagent was used to do out real-time fluorescence quantitative PCR. The expression of GAPDH served as a reference for mRNA expression levels. The 2-ΔΔCq technique was utilized to calculate the relative mRNA expression. The specific primer sequences used are listed in Table S1 of the Supplemental Material.

### 2.5. ELISA assay

Serum was taken from gastric cancer patients and control normal people, and the operation was strictly based on the instructions of the Human Amylin ELISA Kit (Shanghai Enzyme-linked Biotechnology, ml022732). The serum Amylin levels of each group of samples were detected by enzyme labelling instrument (Perkin Elmer, Ensihht, USA).

### 2.6. Immunohistochemical staining

Immunohistochemistry (IHC) was performed on clinical tumour tissues and tumour xenograft specimens using the following primary antibodies: anti-Amylin (1:500 dilution, catalogue #PA5- 84142, Invitrogen), anti-CCN1 (1:100 dilution, catalogue #ab228592, Abcam), anti-Ki67 (1:500 dilution, catalogue #sc-7846, Santa Cruz Biotechnology), anti-E-cadherin (1:200 dilution, catalogue #14472S, Cell Signaling Technology), and anti-N-cadherin (1:200 dilution, catalogue #13116S, Cell Signaling Technology). The tissue sections were deparaffinized in xylene (Sigma-Aldrich, catalogue #Xylene-1) and rehydrated through a graded ethanol series (Sigma-Aldrich, catalogue #E7023). Antigen retrieval was performed by incubating the slides in boiling citrate buffer (pH 6.0, catalogue #C9999, Sigma-Aldrich) for 15 min. Endogenous peroxidase activity was blocked using 3% hydrogen peroxide (catalogue #H1009, Sigma-Aldrich) for 10 min. The sections were then incubated overnight at 4 °C with the primary antibodies. After primary antibody incubation, the slides were washed in phosphate-buffered saline (PBS, catalogue #D8537, Sigma-Aldrich) and incubated for 1 h at room temperature with the corresponding secondary antibody at a dilution of 1:200. Peroxidase activity was visualized using 3,3′-diaminobenzidine (DAB) substrate, and the slides were counterstained with haematoxylin. Images were captured using the 3DHIESTECH scanning system (3DHISTECH, Hungary), and quantitative analysis of antibody expression was performed using Image-Pro Plus 6.0 software (Media Cybernetics Inc., Bethesda, USA). Two independent pathologists, who were blinded to the clinical data and experimental groups, performed the IHC scoring. The average cell-based integrated optical density (IOD) was calculated for each sample, and the final scores were determined by consensus between the two pathologists.

### 2.7. Western blotting

The cells or tissues were disrupted in RIPA buffer (Sigma-Aldrich, Catalogue No. R0278) containing 1% protease inhibitor cocktail (Roche, Catalogue No.04693159001) and 1% phosphatase inhibitor cocktail (Sigma-Aldrich, Catalogue No. P5726) for 30 min on ice. The protein samples were quantified using the BCA protein assay kit (Pierce, Catalogue No. 23227), following the manufacturer’s protocol. Equal amounts of protein (20–30 µg) were separated by sodium dodecyl sulphate-polyacrylamide gel electrophoresis (SDS-PAGE) using a 12% polyacrylamide gel (Bio-Rad, Catalogue No.161-1106) at 120 V for 1.5 h, and then transferred to polyvinylidene fluoride (PVDF) membranes (Millipore, Catalogue No. IPVH00010) using the Trans-Blot Turbo Transfer System (Bio-Rad, Catalogue No.1704150) for 1 h at 200 mA. Following transfer, the membranes were blocked with 5% non-fat dry milk in Tris-buffered saline with 0.1% Tween 20 (TBST) for 1 h at room temperature. Membranes were then incubated with primary antibodies diluted in TBST at optimal concentrations (typically 1:1000 for monoclonal antibodies and 1:2000 for polyclonal antibodies) overnight at 4 °C. After washing with TBST (3 × 10 min), the membranes were incubated with horseradish peroxidase-conjugated secondary antibodies (1:5000 dilution) for 1 h at room temperature. Signal detection was performed using Enhanced Chemiluminescence (ECL) detection reagents (GE Healthcare, Catalogue No. RPN2232), and images were acquired using the Bio-Rad GelDoc XR+ system.

### 2.8. Cell proliferation and colony formation assay

GC cells were seeded in 96-well microtiter plates at a concentration of 1000 cells per well. The cells were incubated for 24, 48, and 72 h, and then treated with Cell Counting Kit-8 (CCK-8, Dojindo Laboratories) at a temperature of 37 °C for a duration of 2 h. The optical density was determined at a wavelength of 450 nm using an ELISA plate reader (BioTek Elx 800, USA). Gastric cancer (GC) cells were introduced into 6-well plates with a concentration of 800 cells per well. Control groups: cells treated with vehicle (PBS); treat groups: cells treated with Amylin as inducer. After a period of 14 days, the colonies were immobilized using a 4% paraformaldehyde solution and then subjected to staining using a 1% crystal violet solution. Photographs were captured and the count of colonies with a diameter bigger than 100 μm was determined.

### 2.9. Edu cell proliferation detection assay

The appropriate number of GC cells were cultured in 6-well plates. Following an overnight culture and restoration to their natural state, the cells were exposed to Amylin therapy. EdU labelling and fixation, washing and permeabilization were handled strictly according to the instructions of the BeyoClick^™^ EdU-647 Cell Proliferation Assay Kit (Beyotime). The images were acquired using a fluorescence microscope manufactured by Leica in Wetzlar, Germany.

### 2.10. Transwell assay

The Transwell test was employed to assess cell migration and invasive potential. In summary, 200 µL of RPMI 1640 cells were placed in the upper chamber of transwell inserts (8 μm, Corning, USA) for migration assays or matrigel-coated transwell inserts for invasion assays. For functional assays, cells were treated with recombinant human amylin or vehicle control (PBS) for 24–48 h. Subsequently, 650 µL of RPMI 1640 containing 10% FBS was added to the lower chamber, regardless of the presence of Stattic. Following a period of 24 or 48 h of incubation, the cells located on the top surface of the implant were extracted using a cotton swab. The migratory or invasive cells were immobilized and subjected to staining. Quantitative analysis was performed on photographs of six randomly selected fields from three duplicate wells.

### 2.11. Apoptosis assay

The GC cells were rinsed twice, then mixed with 100 μl of 1 × binding buffer that included 5 μl of AnnexinV-FITC and 5 μl of PI staining solution. The mixture was then left to incubate in the dark for 30 min. The samples were combined with 400 μl of 1 × Binding Buffer and analysed using flow cytometry (BD Biosciences, USA).

### 2.12. In vivo growth assays

Purchased from Shulaibao Biotechnology (Wuhan, China), male BALB/c nude mice aged 4-6 weeks were obtained. HGC-27 cells (2 × 10^6^) treated with either Amylin (100ug/kg) or CCN1-overexpressing lentivirus were injected under the skin of nude mice on the lateral wall, control groups treated with PBS. The mice were euthanized by cervical dislocation three weeks after injection, and the tumors were surgically removed and their weight was measured. The estimation of tumor volume was calculated using the formula: volume = length × width^2^/2. The excised tumors were either placed in paraffin for immunohistochemical examination or immediately frozen in liquid nitrogen for protein extraction.

### 2.13. Next-generation sequencing technology (NGS)

For transcriptomic analysis, RNA sequencing (RNA-seq) was performed on the gastric cancer cell lines. Total RNA was extracted using the RNAiso Plus reagent (catalogue #9108, Takara, Japan) according to the manufacturer’s instructions. RNA quality and quantity were assessed using the NanoDrop 2000 (Thermo Fisher Scientific, USA) and the Agilent 2100 Bioanalyzer (Agilent Technologies, USA). Sequencing was performed on the Illumina NovaSeq 6000 platform (Illumina, USA) with paired-end 150 bp reads. A minimum of 30 million reads per sample was targeted for each sequencing run. The raw sequencing data were processed using the FASTQC tool (version 0.11.9) to assess read quality, followed by trimming and filtering using Trimmomatic (version 0.39) to remove adapter sequences and low-quality bases. Clean reads were aligned to the hg38 reference genome using STAR (version 2.7.3a) aligner. Gene expression levels were quantified by HTSeq (version 0.11.2) using the Ensembl gene annotation (release 102). Differential gene expression analysis was performed using DESeq2 (version 1.30.1), with genes having a false discovery rate (FDR) < 0.05 considered significantly differentially expressed. Functional enrichment analysis was conducted using Kyoto Encyclopedia of Genes and Genomes (KEGG) pathway analysis through the clusterProfiler R package (version 3.18.1).

### 2.14. Data handling and statistical analysis

All experiments were performed with at least three independent biological replicates to ensure the reliability and reproducibility of the results. Outliers were identified using the interquartile range (IQR) method, and if confirmed to be due to technical errors, they were excluded. Missing data were handled by excluding minimal missing values (<5%) or using multiple imputation methods for larger amounts of missing data to maintain statistical power and validity. The statistical analysis was conducted using SPSS software (version 21.0, IBM Corp, Armonk, NY, USA). The values are shown as the average ± standard deviation of a minimum of three separate trials. The comparison of quantitative variables was conducted using Student’s t-tests and Mann–Whitney U-tests, when appropriate. Furthermore, a unidirectional analysis of variance (ANOVA) was conducted, followed by a Turkish *post hoc* test, to compare various groups. The analysis of qualitative variables was conducted using either Pearson χ2 or Fisher exact tests. Log-rank tests were used to compare Kaplan-Meier curves. A significance level of *p* < 0.05 was used to determine statistical significance.

## 3. Results

### 3.1. Expression of amylin in gastric cancer tissues and serum

**Figure 1.**
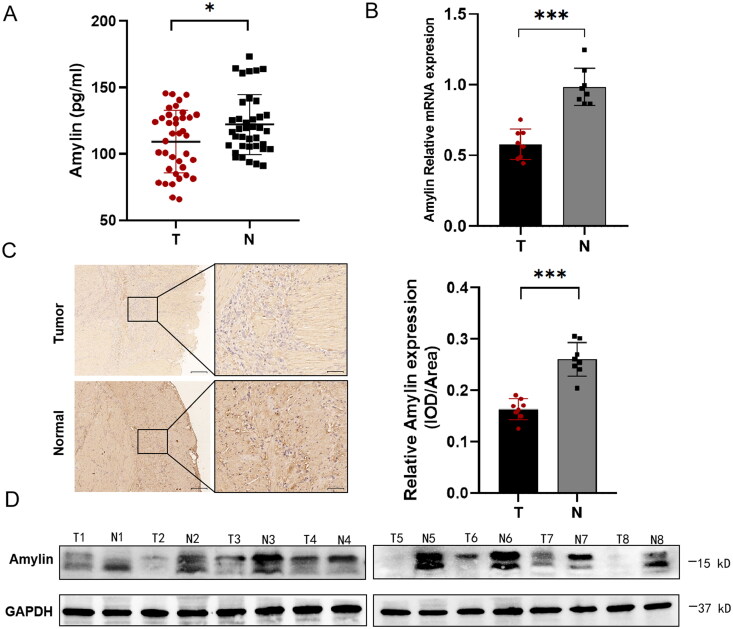
Amylin expression in serum and tissues of gastric cancer group and normal control group. (A) Amylin expression in serum of normal and gastric cancer patients (B) Amylin mRNA expression in 8 pairs of gastric cancer and their surrounding normal tissues was verified using qPCR. (C) Representative images of immunohistochemical experiments with amylin in 8 pairs of gastric cancer and adjacent normal tissues and the distribution of mean IOD levels in the samples. Scale bars, 200 μm (left panel) or 50 μm (right panel). (D) Western blotting was used to assess the amounts of amylin protein in 8 pairs of stomach cancer and surrounding normal tissues. (*, *p* < 0.05; **, *p* < 0.01; ***, *p* < 0.001).

By examining the expression levels of Amylin in the serum of 38 pairs of gastric cancer patients and normal subjects, we found that Amylin was down-regulated in the serum of gastric cancer patients (Figure 1(A)). A qPCR test conducted on 8 pairs of gastric cancer and adjacent non-cancerous tissues confirmed significantly reduced Amylin mRNA expression in gastric cancer tissues (Figure 1(B)). Immunohistochemical labelling further demonstrated a significant under-expression of Amylin protein in gastric cancer tissues compared to nearby normal gastric tissues (Figure 1(C)). Additionally, Western blot analysis confirmed the reduced expression of Amylin protein in gastric cancer tissues (Figure 1(D)).

### 3.2. Amylin expression and clinical characterization

**Figure 2.**
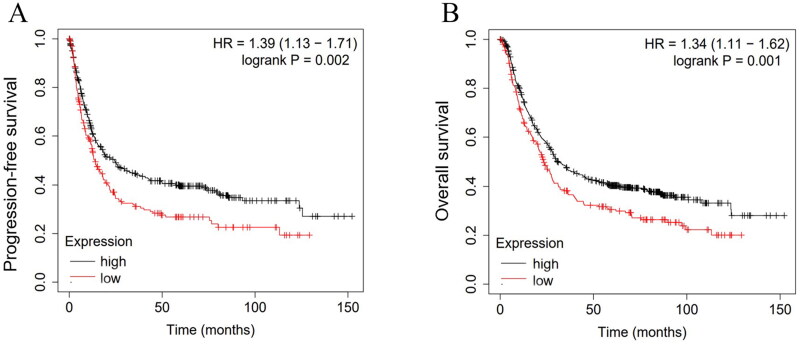
The relationship between the expression of amylin and the prognosis of patients with gastric cancer. (A) The Kaplan–Meier curves demonstrate the progression-free survival rates of 875 gastric cancer patients from the GSE database, categorized based on their high or low amylin expression levels, *p* = 0.002. (B) The Kaplan–Meier curves demonstrate the overall survival of gastric cancer patients in the GSE database, *p* = 0.001.

In this experiment, the correlation between Amylin levels and various pathological parameters in gastric cancer patients was analysed based on serum Amylin expression in 38 patients. The findings demonstrated that the patients’ age, sex, degree of differentiation, lymphatic metastasis, and distant metastasis did not significantly correlate with Amylin expression (*p* > 0.05). However, high Amylin expression was negatively correlated with tumour diameter (*p* = 0.003, 95% CI: 0.371 to 1.763), TNM stage (*p* = 0.010, 95% CI:0.212 to 1.548), and deep tumour infiltration (*p* = 0.042, 95% CI:0.025 to 1.434), as shown in Table 1. Additionally, analysis of six GSE datasets (GSE14210, GSE15459, GSE22377, GSE29272, GSE51105, GSE62254) involving 875 gastric cancer patients revealed that those with high Amylin expression had significantly longer progression-free survival (*p* = 0.002) and overall survival (*p* = 0.001) compared to patients with low Amylin expression, as illustrated in Figure 2(A,B).

**Table 1.** The relationship between the expression of amylin and the clinicopathologic characteristics in gastric cancer patients.

Clinical Features	Number	Amylin (pg/ml)	*t*/F	Pearson	*P* value
Age(years)			0.973	0.027	0.337
>60	16	117.30			
≤60	22	113.10			
Sex			0.070	−0.012	0.945
Male	25	114.00			
Female	13	114.68			
Tumor size (cm)			3.234	**−0.474**	**0.003**
>3	23	103.55			
≤3	15	130.61			
Cell differentiation			1.380	0.122	0.265
Well	3	104.82			
Moderate	15	123.48			
Poor	20	108.71			
TNM staging			2.730	**−0.414**	**0.010**
I-II	19	125.77			
III-IV	19	102.69			
Tumor invasion depth			2.104	**−0.331**	**0.042**
T1-T2	12	127.81			
T3-T4	26	107.97			
Lymph node metastasis			0.827	−0.136	0.413
No	15	118.94			
Yes	23	111.16			
Distance Metastasis			1.470	−0.238	0.150
M0	33	116.81			
M1	5	97.20			

### 3.3 Effect of amylin on the proliferative capacity of gastric cancer cells

**Figure 3.**
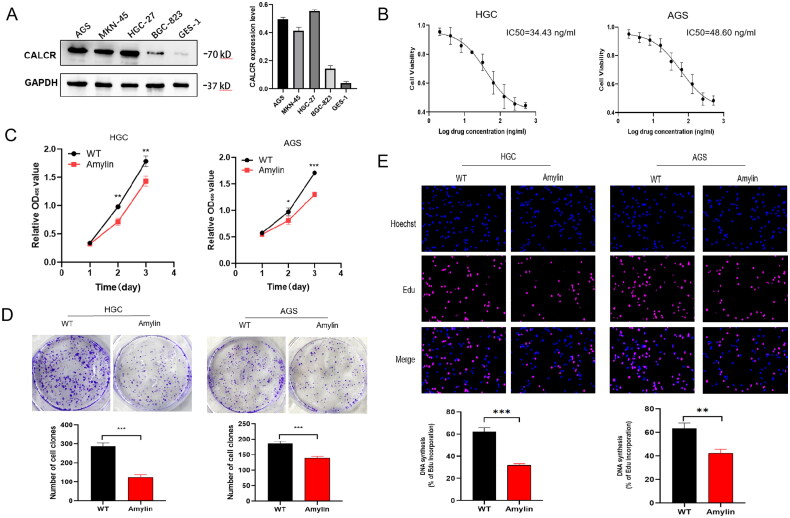
The impact of amylin on the growth and multiplication of gastric cancer cells. (A) Western blotting was used to identify the expression levels of the amylin receptor CALCR in different gastric cancer cells. (B) The IC50 of the optimal dosing concentration of amylin in HGC and AGS cell lines was detected by CCK8 assay. (C) The impact of amylin on the survival of the specified stable cells was assessed using the CCK-8 test. (D) a clone formation test was conducted to assess the impact of amylin on the capacity of gastric cancer cells to generate clones. The upper panel displays a typical image of the colonies, while the below panel quantifies the number of colonies. (E) Edu cell proliferation test was conducted to evaluate the impact of amylin on the activity of cellular DNA replication. (*, *p* < 0.05; **, *p* < 0.01; ***, *p* < 0.001).

In order to investigate the role of Amylin in gastric cancer cells, we collected proteins from both normal gastric epithelial cells (GES-1) and gastric cancer cells (AGS, MKN-45, HGC-27, BGC-823). We then examined the presence of the Amylin receptor CALCR and found it significantly more abundant in most gastric cancer cell lines, particularly in AGS and HGC-27 (Figure 3(A)). Consequently, we selected AGS and HGC cell lines for subsequent experiments. Using the CCK-8 assay, we determined the optimal dosing concentration of Amylin to be 34.43 ng/ml for HGC cells and 48.60 ng/ml for AGS cells (Figure 3(B)). We used these concentrations in further experiments. The CCK-8 test revealed that Amylin notably inhibited the growth and division of HGC and AGS cells compared to the control group (Figure 3(C)). Additionally, a plate cloning assay demonstrated that Amylin inhibited clone formation (Figure 3(D)), and an Edu cell proliferation assay indicated that Amylin reduced DNA replication activity in gastric cancer HGC and AGS cells (Figure 3(E)). These findings suggest that Amylin inhibits gastric cancer HGC and AGS cells proliferation in both long-term and short-term processes.

### 3.4. Effect of amylin on migration and invasion ability of gastric cancer cells

**Figure 4.**
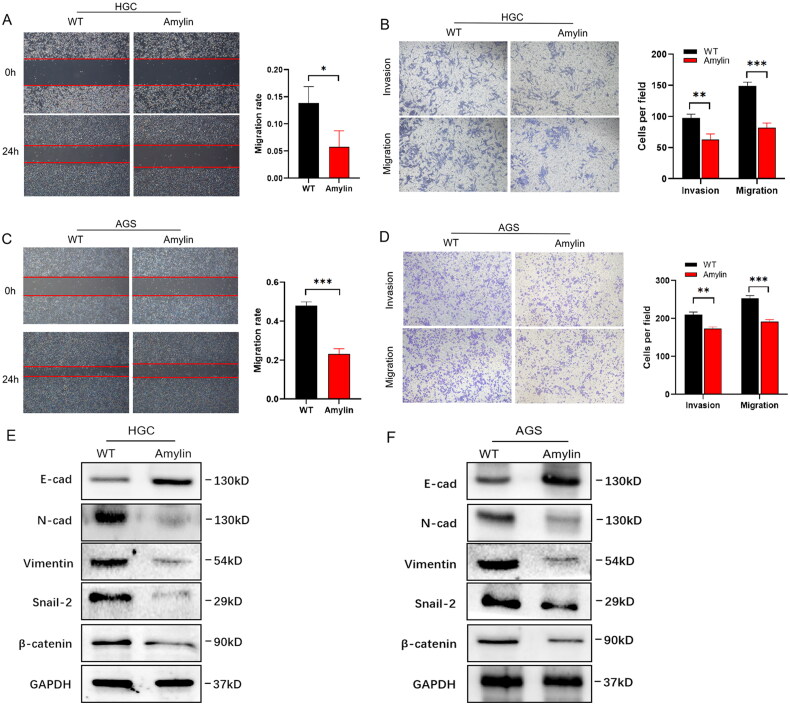
Effect of amylin on cell migration and invasion. (A) Cell migration of amylin-added HGC cells was determined by scratch assay at 24h (0h) after scratching. Scale bar, 200 μm.(B) The Transwell experiment was used to examine the cell migration and invasion capabilities of HGC cells following the addition of amylin. The left side displays representative photos, the right side presents bar graphs that provide a summary of the analysis. Scale bar, 50 μm.(C) Cell migration of amylin-added AGS cells was determined by scratch assay at 24h (0h) after scratching. Scale bar, 200 μm.(D) The cell migration and invasion capacity of AGS cells with the addition of amylin were assessed using the transwell test. The left side displays representative photos, while the right side presents bar graphs that provide a summary of the analysis. Scale bar, 50 μm. (E and F) The presence of EMT markers, including E-cadherin, N-cadherin, Vimentin, snail-2, and β-catenin, was identified in the cells using Western blotting. (*, *p* < 0.05; **, *p* < 0.01; ***, *p* < 0.001).

To evaluate the impact of Amylin on cell metastasis, we performed a scratch assay. The results indicated that Amylin significantly inhibited the migration rate of HGC and AGS gastric cancer cells (Figure 4(A,C)). Transwell assays corroborated these findings, showing that Amylin inhibited the invasive and migratory capacities of HGC and AGS cells (Figure 4(B,D)). Considering that epithelial-mesenchymal transition (EMT) is a characteristic feature of increased cellular activity and metastatic potential, we examined the levels of several EMT markers. Figure 4(E,F) shows that Amylin increased the expression of the cell surface protein E-cadherin and decreased the levels of EMT markers N-cadherin, Vimentin, Snail-2, and β-catenin. These results suggest that Amylin regulates the EMT process. In conclusion, our findings demonstrate that Amylin plays a crucial role in controlling cell movement and invasion and modulates the EMT process in gastric cancer cells.

### 3.5. Amylin targets downstream CCN1 gene

**Figure 5.**
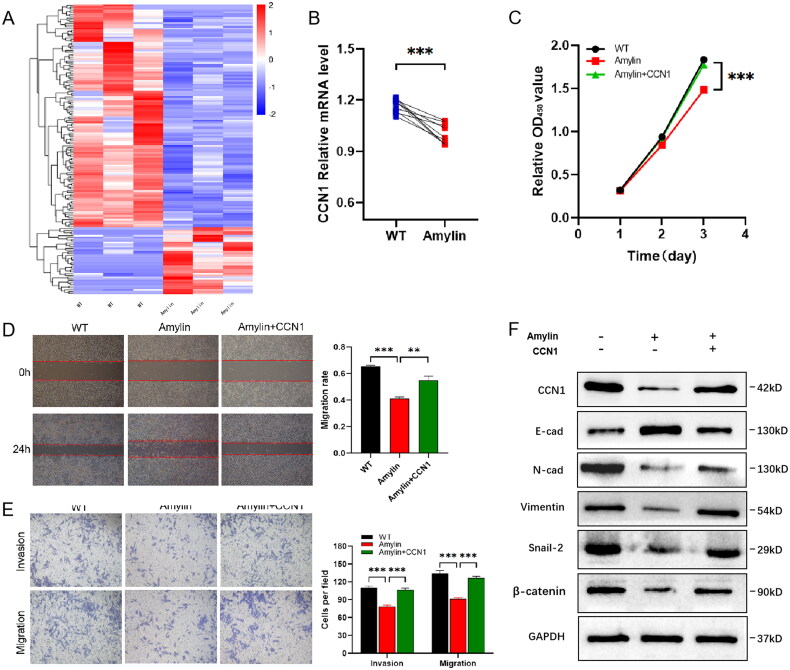
Amylin affects gastric cancer HGC cell function by targeting CCN1. (A) Detection of the effect of amylin on transcriptome gene expression in gastric cancer HGC cells by transcriptome sequencing. (B) The effect of amylin on CCN1 gene expression was verified by qPCR assay. (C) The alteration of cell proliferation ability induced by amylin could be replied after overexpression of CCN1 was detected by CCK-8. (D) The ability to revert to amylin-induced changes in cell migration ability after overexpression of CCN1 was detected by scratch assay. Scale bar, 200 μm. (E) The effects of amylin on cell migration and invasion were observed using the transwell test following the overexpression of CCN1. Scale bar, 50 μm. (F) Changes in EMT index of cells induced by the high expression of CCN1 under amylin. (**, *p* < 0.01; *** *p* < 0.001).

To gain deeper insights into the mechanism by which Amylin affects gastric cancer cells, we subjected Amylin-treated gastric cancer HGC cells and control HGC cells to second-generation transcriptome sequencing analysis. We screened for differentially expressed genes using the criteria of a fold change (Foldchange) ≥ 2 and a false discovery rate (FDR) < 0.05. Our findings indicated an increase in the expression of 37 genes and a decrease in the expression of 124 genes in the experimental group compared to the control group (Figure 5(A)). Notably, the most significantly down-regulated gene was CCN1 (Table S2). *In vitro* experiments confirmed that Amylin significantly downregulated CCN1 gene expression (Figure 5(B)). Further experiments involving the overexpression of CCN1 revealed a dramatic reversal of the suppression of HGC cell growth (Figure 5(C)), migration, and invasion caused by Amylin (Figure 5(D,E)). The expression of several EMT markers affected by Amylin was also restored (Figure 5(F)). These results demonstrated that Amylin modulates the proliferation, migration, and invasion of gastric cancer cells by targeting CCN1.

### 3.6. Amylin inhibits activation of the PI3K/AKT signalling pathway

**Figure 6.**
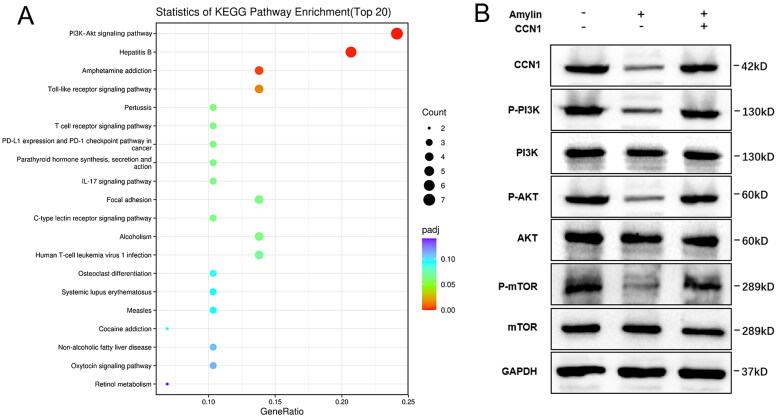
Amylin was significantly enriched with PI3K/AKT pathway. (A) Amylin caused enrichment analysis of KEGG pathway of differentially expressed genes in gastric cancer cells. (B) Performing a Western blotting test to confirm the impact of amylin on the PI3K/AKT signalling pathway.

Enrichment analysis of KEGG signalling pathways was performed on the differentially expressed genes. The analysis revealed that these genes were involved in 35 classical signalling pathways (Table S3). Among these pathways, the PI3K/AKT signalling pathway was notably enriched. The effect of Amylin on the PI3K/AKT pathway was assessed using Western blotting. Results showed that the relative expression of PI3K, AKT, and mTOR proteins in HGC gastric cancer cells remained relatively stable after the introduction of Amylin. However, the relative expression levels of p-PI3K, p-AKT, and p-mTOR proteins were significantly lower compared to the control group, with statistical significance (*p* < 0.05) as illustrated in Figure 6.

### 3.7. Amylin inhibits tumour development in nude mice in vivo

**Figure 7.**
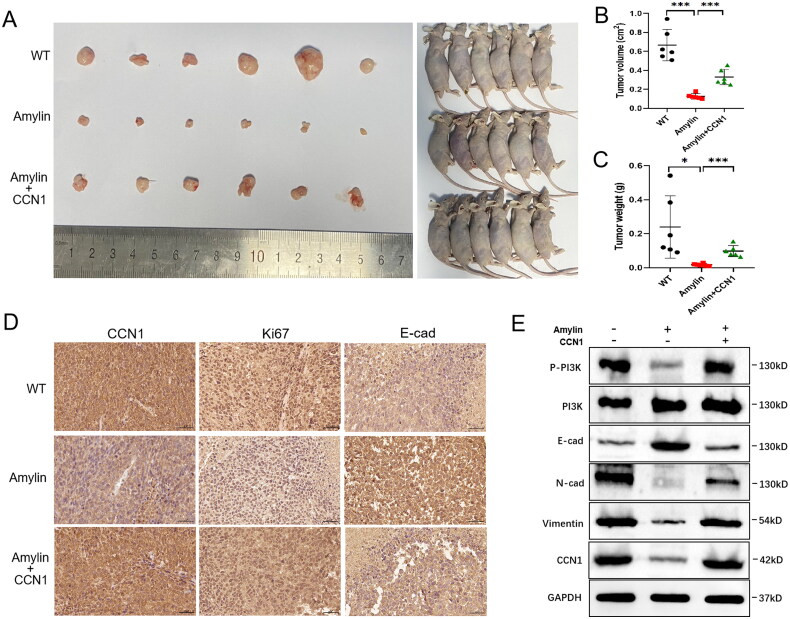
Amylin inhibits tumorigenicity in nude mice. (A) Stably transfected control or CCN1 overexpressing HGC cells were injected into the flanks of nude mice. Once the tumour reached a diameter of 3-5 mm, HGC cells were subjected to amylin (100 ug/kg per day for 10-12 days) treatment. Photographs of xenograft tumours isolated 21 days after inoculation in nude mice. (B) At the end of the experiment, tumour volumes of the three groups were compared. (C) Tumour weights of the three groups were compared. (D) Immunohistochemical (IHC) detection of CCN1, Ki67, and E-cadherin expression in the subcutaneous xenografts as indicated. Scale bar, 50 μm. (E) Western blot analysis was conducted to measure the levels of P-PI3K, PI3K, E-cadherin, N-cadherin, vimentin, and CCN1 in three representative xenograft tumours from each group. (*p < 0.05, **p < 0.01, ***p < 0.001.).

The involvement of Amylin in the progression of gastric cancer was further investigated using *in vivo* models. Figure 7(A–C) shows that Amylin significantly suppressed tumour volume growth and tumour weight increase in the intramuscular xenograft model. However, this inhibitory effect was abrogated by CCN1 overexpression. Immunohistochemistry confirmed that Amylin inhibited CCN1 expression in xenograft tumours. Compared to the control group, Amylin markedly inhibited cell proliferation, as indicated by Ki-67 staining. Furthermore, E-cadherin expression was significantly elevated in the Amylin-treated xenografts. Western blotting verified that Amylin decreased the expression of P-PI3K, vimentin, and N-cadherin. Overexpression of CCN1 reversed all these effects (Figure 7(D,E)). In conclusion, the *in vivo* experiments validated the molecular pathways identified *in vitro*.

## 4. Discussion

The formation, progression, and metastasis of gastric cancer (GC) is a complex process influenced by various factors, including genetic variation, cell growth and proliferation, angiogenesis, infiltration, vascular embolization, and apoptotic cell survival and evasion [21]. Tumour survival, growth, and metastasis largely depend on interactions with the microenvironment [22]. Amylin, also known as islet amyloid polypeptide (IAPP), is a neuroendocrine hormone with diverse biological functions, including the regulation of energy balance, signalling obesity, controlling bone metabolism, regulating blood pressure, and overseeing exercise activity [6,23]. Amylin is the third most significant hormone produced by islet cells, following insulin and glucagon [8]. While insulin and glucagon have established roles in cancer progression, the relationship between amylin and tumours remains unclear [24–26]. Previous studies have found that soluble factors in pancreatic cancer cells selectively stimulate the secretion of Amylin from pancreatic islet cells, and that elevated Amylin concentrations are an early feature of pancreatic cancer [11]. Overall studies on the association of Amylin with other tumors are still very rare, especially in gastric cancer. Studies focusing on amylin provide new insights into gastric carcinogenesis and progression, presenting opportunities to explore biomarkers and therapeutic targets for gastric cancer.

In this study, the effect of Amylin on gastric cancer was investigated for the first time. Results indicated that Amylin expression levels were significantly reduced in the serum and tissues of gastric cancer patients compared to normal controls. Furthermore, additional experimental findings revealed a correlation between Amylin expression levels and patient survival rates in gastric cancer. High Amylin expression was inversely correlated with tumour size, stage, and depth of infiltration, all of which are unfavourable pathological characteristics in clinical settings for gastric cancer patients. These results suggest that Amylin may have prognostic value and a protective role in the onset and progression of gastric cancer. One of the main reasons for poor treatment outcomes in gastric cancer patients is metastasis [27]. EMT is a crucial molecular step in metastasis [28]. As the initial stage of the metastatic process, EMT involves the disassembly of intercellular junctions and the loss of apical-basal polarity [29]. During EMT, gastric cancer cells downregulate cell adhesion proteins (e.g. E-cadherin) and tight junction proteins while upregulating mesenchymal markers (e.g. N-cadherin) [30]. Understanding the mechanisms underlying EMT in gastric cancer metastasis could facilitate the development of specific therapeutic strategies. Consequently, we conducted further investigations into the biological activities and processes involving Amylin in gastric cancer. Our findings demonstrated a strong inhibitory effect of Amylin on the proliferation of gastric cancer cells. Additionally, we found that Amylin played a key role in regulating cell movement and infiltration, and modulating the EMT process in gastric cancer cells. Specifically, the addition of Amylin to gastric cancer cells increased the expression of the cell surface protein E-cadherin and inhibited the EMT markers N-cadherin, Vimentin, Snail-2, and β-catenin. Further studies confirmed that the changes in EMT observed in tumour tissues formed subcutaneously in nude mice were consistent with *in vitro* results. This implies that by regulating the EMT process, Amylin may influence the biological activity of gastric cancer cells.

Gastric cancer is a multifaceted process involving multiple steps and influenced by genetic and epigenetic alterations. These alterations lead to the activation of proto-oncogenes, inactivation of tumor suppressor genes, and the subsequent loss of regulation over normal cellular functions [31]. This study utilized Next-Generation Sequencing technology to investigate the impact of Amylin on gene activity in gastric cancer. Results indicated that Amylin upregulated 37 genes and downregulated 124 genes, with the CCN1 gene being the most significantly downregulated. A CCN1 overexpression assay demonstrated that increased CCN1 expression effectively counteracted the suppression of HGC cell proliferation, migration, and invasion induced by Amylin. Furthermore, CCN1 overexpression reversed changes in the expression of various EMT markers induced by Amylin. These findings suggest that Amylin may contribute to gastric cancer progression by modulating CCN1 gene expression. Existing studies have shown that CCN1 is correlated with various signaling pathways. Li et al. ‘s study showed that CCN1 is a direct target of Wnt/β-catenin signal transduction in hepatocellular carcinoma [32]; Niu et al.’ s study found that CCN1 is a tumor promoter in acute myeloid leukemia (AML). c-Myc and Bcl-xL are up-regulated and Bax down-regulated through the MEK/ERK pathway [33]. In addition, CCN1 stimulates the expression of PI3K/AKT in a variety of tumour cells, including glioma, breast cancer, gastric cancer and kidney cancer [18–20]. The PI3K/Akt signalling pathway, a critical intracellular signaling mechanism associated with cell quiescence, division, cancer, and longevity, is currently a major focus of research [34]. Upregulation of the PI3K/Akt pathway and glycolysis enhances glucose consumption, providing an evolutionary advantage to both normoxic and hypoxic cancer cells [35]. The PI3K/Akt pathway is a well-known oncogenic pathway implicated in various cancers, including breast cancer, prostate cancer, and glioblastoma [36–38]. Our study found that Amylin and PI3K/Akt signalling pathway is significantly enriched in gastric cancer. CCN1 induced the expression of the PI3K/Akt pathway in gastric cancer cells, counteracting the effect of Amylin on this pathway. These results suggest that Amylin may influence gastric cancer progression by regulating the PI3K/Akt signalling pathway. Amylin significantly inhibited the expression of the downstream CCN1 gene and suppressed the PI3K/AKT signalling pathway, thereby affecting gastric cancer development and progression.

The findings of this study have significant clinical implications for the treatment of gastric cancer. The downregulation of Amylin in gastric cancer tissues and its inverse correlation with adverse pathological features suggest that Amylin could serve as a valuable prognostic biomarker. Patients with low Amylin expression may benefit from targeted therapies aimed at restoring Amylin levels or enhancing its activity. Amylin’s ability to inhibit tumour growth, migration, and invasion, as well as its role in modulating the EMT process, highlights its potential as a therapeutic agent. Amylin analogues, such as pramlintide, which is already approved for the treatment of diabetes, could be repurposed for gastric cancer therapy. Preclinical studies should explore the efficacy of Amylin analogues in combination with existing chemotherapeutic agents to enhance treatment outcomes. Our findings also implicate CCN1 as a key mediator of Amylin’s anti-tumour effects. CCN1 inhibitors, either alone or in combination with Amylin, could provide a novel therapeutic strategy for gastric cancer.

## 5. Limitation

While our study provides novel insights into the role of Amylin in gastric cancer, certain limitations should be acknowledged to guide future research. Firstly, the clinical cohort included 38 gastric cancer patients, which, while sufficient for initial exploratory analysis, may benefit from expansion in larger, multi-centre studies to enhance statistical power and generalizability. Secondly, although we utilized multiple gastric cancer cell lines and an *in vivo* xenograft model, these experimental systems may not fully capture the complexity of human gastric cancer. Future studies could incorporate patient-derived xenografts or organoid models to better mimic the tumour microenvironment. Thirdly, while RNA sequencing provided valuable insights into Amylin-regulated genes and pathways, the analysis was based on a limited number of samples. Expanding the sample size and integrating multi-omics approaches could further elucidate the molecular mechanisms underlying Amylin’s effects.

## 6. Conclusion

In summary, this study is the first to demonstrate that Amylin exerts anti-tumour effects in gastric cancer by targeting CCN1 and inhibiting the PI3K/Akt pathway. Our findings provide a new mechanistic understanding of how Amylin, a neglected hormone associated with glucose metabolism, can influence cancer progression. This opens up potential therapeutic avenues for targeting Amylin and its downstream effectors in gastric cancer treatment.

## Supplementary Material

Supplemental Material

## Data Availability

The datasets used and/or analysed during the current study are available from the corresponding author on reasonable request.
