# Supplementary material for: Amylin inhibits gastric cancer progression by targeting CCN1 and affecting the PI3K/AKT signalling pathway
Source: Ann Med. 2025 Mar 31;57(1):2480754. doi: 10.1080/07853890.2025.2480754 (PMC12931309; doi:10.1080/07853890.2025.2480754)
Supplement: Supplemental Material [file IANN_A_2480754_SM3291.zip › Suppl/Table_S1.docx]

| Primer names | Sequences （5’→3’） | | Lengths（bp） | Amplicon Size (bp) |
| --- | --- | --- | --- | --- |
| CCN1-F | | TTCCAGGGCACACCTAGACAAAC | 23 | 96 |
| CCN1-R | | ACAATGAGTCCCATCACCCACAC | 23 | 96 |
| Amylin-F | | GGAATGGCTAGTTCCGGTTTTGC | 23 | 141 |
| Amylin-R  GAPDH-F  GAPDH-R | | AGGTGTTGAGTCGCACAAGTGG  ACCACAGTCCATGCCATCAC  TCCACCACCCTGTTGCTGTA | 22  20  20 | 141  108  108 |

Table S1 Sequences of primers used for PCR in this study
