# Supplementary material for: Amylin inhibits gastric cancer progression by targeting CCN1 and affecting the PI3K/AKT signalling pathway
Source: Ann Med. 2025 Mar 31;57(1):2480754. doi: 10.1080/07853890.2025.2480754 (PMC12931309; doi:10.1080/07853890.2025.2480754)
Supplement: Supplemental Material [file IANN_A_2480754_SM3291.zip › Suppl/Table_S2.docx]

Table S2 Top 10 differentially expressed genes down-regulated and up-regulated by Amylin in gastric cancer cells

| Gene Symbol | log2FoldChange | *P* value | Regulation |
| --- | --- | --- | --- |
| CCN1  CCDC180  H2BC21  ARC  FOSB  LOC124901232  EGR3  LOC124904652  FOS  IER2 | -1.053  -2.185  -1.355  -1.394  -1.357  -1.144  -1.804  -1.420  -2.320  -1.293 | 5.34E-19  8.47E-17  8.22E-14  1.66E-11  1.20E-09  1.63E-08  4.62E-08  3.28E-07  3.46E-07  2.18E-06 | Down  Down  Down  Down  Down  Down  Down  Down  Down  Down |
| TM4SF19  SENP3-EIF4A1  HSPE1-MOB4  CORIN  LOC124904926  LOC102723834  LOC105377412  CD300A  BLOC1S5-TXNDC5  PDE6B | 4.356  1.626  1.248  4.598  1.622  4.487  4.497  4.522  3.361  4.428 | 0.00208  0.00282  0.00710  0.00875  0.00993  0.01196  0.01221  0.01273  0.01329  0.01447 | Up  Up  Up  Up  Up  Up  Up  Up  Up  Up |
