# Supplementary material for: Amylin inhibits gastric cancer progression by targeting CCN1 and affecting the PI3K/AKT signalling pathway
Source: Ann Med. 2025 Mar 31;57(1):2480754. doi: 10.1080/07853890.2025.2480754 (PMC12931309; doi:10.1080/07853890.2025.2480754)
Supplement: Supplemental Material [file IANN_A_2480754_SM3291.zip › Suppl/Table_S3.docx]

**Table S3** KEGG pathway enrichment of Amylin in gastric cancer HGC cells

| Serial No. | Name of pathway | *P* value |
| --- | --- | --- |
| \| 1 \| \| --- \| \| 2 \| \| 3 \| \| 4 \| \| 5 \| \| 6 \| \| 7 \| \| 8 \| \| 9 \| \| 10 \| \| 11 \| \| 12 \| \| 13 \| \| 14 \| \| 15 \| \| 16 \| \| 17 \| \| 18 \| \| 19 \| \| 20 \| \| 21 \| \| 22 \| \| 23 \| \| 24 \| \| 25 \| \| 26 \| \| 27 \| \| 28 \| \| 29 \| \| 30 \| \| 31 \| \| 32 \| \| 33 \| \| 34 \| \| 35 \| | \| PI3K-Akt signaling pathway \| \| --- \| \| Hepatitis B \| \| Amphetamine addiction \| \| Toll-like receptor signaling pathway \| \| Pertussis \| \| PD-L1 expression and PD-1 checkpoint pathway \| \| Alcoholism \| \| IL-17 signaling pathway \| \| Focal adhesion \| \| C-type lectin receptor signaling pathway \| \| T cell receptor signaling pathway \| \| Parathyroid hormone synthesis, secretion and action \| \| Human T-cell leukemia virus 1 infection \| \| Osteoclast differentiation \| \| Systemic lupus erythematosus \| \| Measles \| \| Cocaine addiction \| \| Non-alcoholic fatty liver disease \| \| Oxytocin signaling pathway \| \| Retinol metabolism \| \| Renal cell carcinoma \| \| Kaposi sarcoma-associated herpesvirus infection \| \| Leishmaniasis \| \| B cell receptor signaling pathway \| \| Chemical carcinogenesis \| \| ErbB signaling pathway \| \| Colorectal cancer \| \| Human immunodeficiency virus 1 infection \| \| Salmonella infection \| \| Regulation of actin cytoskeleton \| \| Th1 and Th2 cell differentiation \| \| GnRH signaling pathway \| \| Rheumatoid arthritis \| \| Endocrine resistance \| \| Choline metabolism in cancer \| | \| \| 6.64E-06 \| \| --- \| \| 2.17E-05 \| \| 0.000107216 \| \| 0.000521859 \| \| 0.002609169 \| \| 0.004082363 \| \| 0.004537485 \| \| 0.004759760 \| \| 0.005859077 \| \| 0.006309531 \| \| 0.006309531 \| \| 0.006651565 \| \| 0.007909031 \| \| 0.010917546 \| \| 0.013135593 \| \| 0.013661093 \| \| 0.013743185 \| \| 0.016765999 \| \| 0.018298404 \| \| 0.024124097 \| \| 0.026207073 \| \| 0.031170215 \| \| 0.031339949 \| \| 0.036030475 \| \| 0.036837352 \| \| 0.038472107 \| \| 0.039299869 \| \| 0.041669359 \| \| 0.042162065 \| \| 0.042657761 \| \| 0.044408197 \| \| 0.045282681 \| \| 0.045282681 \| \| 0.048844555 \| \| 0.049750734 \| \| \| --- \| --- \| --- \| --- \| --- \| --- \| --- \| --- \| --- \| --- \| --- \| --- \| --- \| --- \| --- \| --- \| --- \| --- \| --- \| --- \| --- \| --- \| --- \| --- \| --- \| --- \| --- \| --- \| --- \| --- \| --- \| --- \| --- \| --- \| --- \| --- \| |
|  |  |  |
